# Supplementary material for: Prediction of iodine-125 seed implantation efficacy in lung cancer using an enhanced CT-based nomogram model
Source: PLoS One. 2024 Nov 15;19(11):e0313570. doi: 10.1371/journal.pone.0313570 (PMC11567524; doi:10.1371/journal.pone.0313570)
Supplement: Attachment — Submitted filename: Response to Reviewers.doc [file pone.0313570.s001.doc]

**Comments from the editors and reviewers:**

**-Editor**

**When submitting your revision, we need you to address these additional requirements.**

**1. Please ensure that your manuscript meets PLOS ONE's style requirements, including those for file naming. The PLOS ONE style templates can be found at https://journals.plos.org/plosone/s/file?id=wjVg/PLOSOne_formatting_sample_main_body.pdf and** [**https://journals.plos.org/plosone/s/file?id=ba62/PLOSOne_formatting_**](https://journals.plos.org/plosone/s/file?id=ba62/PLOSOne_formatting_)**sample_title_**

**authors_affiliations.pdf**

**Response**: Thank you for your suggestion. I have reviewed the provided templates and ensured that our manuscript now fully complies with the PLOS ONE style requirements, including the file naming conventions. We have standardized the writing format of the article title, all first-level headings, second-level headings, and their subheadings, using sentence case except for proper nouns and the first letter. In addition, we have standardized the affiliation information by removing the postal codes. Lastly, we have standardized the corresponding author information by placing the corresponding author's designation in parentheses with the first letter capitalized.

**2.** **Please provide additional details regarding participant consent. In the ethics statement in the Methods and onLine submission information, please ensure that you have specified (1) whether consent was informed and (2) what type you obtained (for instance, written or verbal, and if verbal, how it was documented and witnessed). If your study included minors, state whether you obtained consent from parents or guardians. If the need for consent was waived by the ethics committee, please include this information.If you are reporting a retrospective study of medical records or archived samples, please ensure that you have discussed whether all data were fully anonymized before you accessed them and/or whether the IRB or ethics committee waived the requirement for informed consent. If patients provided informed written consent to have data from their medical records used in research, please include this information.**

**Response:** Thank you for your suggestion. We have made additions to the ethics statement in the Methods section of the manuscript: Our study is a retrospective analysis using medical record data that was approved in writing by the Yichang Central People's Hospital Ethics Committee. According to the review by the Ethics Committee, the requirement for patient consent was waived, as all data were fully anonymized before we accessed them and did not contain any personally identifiable information. Since this study did not involve minors, there was no need to obtain consent from parents or guardians.The corresponding revision is on **Page 5, Line 83-86**.

**3. Thank you for stating the following financial disclosure: “the Open Fund of Hubei Provincial Key Laboratory of Molecular Imaging (No. 2023fzyx024) and the Hubei Provincial Traditional Chinese Medicine Research Project (No. ZY2023M038)”. Please state what role the funders took in the study. If the funders had no role, please state: "The funders had no role in study design, data collection and analysis, decision to publish, or preparation of the manuscript."If this statement is not correct you must amend it as needed. Please include this amended Role of Funder statement in your cover letter; we will change the onLine submission form on your behalf.**

**Response:** Thank you for your suggestion. The revised role of funder statement is as follows:The funders played a role in study design, data collection, analysis, and manuscript review, but had no role in the decision to publish or manuscript preparation. We have included this revised statement in the cover letter and kindly request you to update the onLine submission form accordingly.

**4. In the onLine submission form you indicate that your data is not available for proprietary reasons and have provided a contact point for accessing this data. Please note that your current contact point is a co-author on this manuscript. According to our Data Policy, the contact point must not be an author on the manuscript and must be an institutional contact, ideally not an individual. Please revise your data statement to a non-author institutional point of contact, such as a data access or ethics committee, and send this to us via return email. Please also include contact information for the third party organization, and please include the full citation of where the data can be found.**

**Response:** Thank you for your suggestion. In accordance with PLOS’s data policy requirements, we have changed the data access contact point to a non-author institutional contact. The data can be accessed through the Ethics Committee of our institution, with the following contact details: [Yichang Central People's Hospital Ethics Committee, shenxl2022@163.com]. We have updated the data availability statement in the manuscript and have already sent this information to you via email.

**5. PLOS requires an ORCID iD for the corresponding author in Editorial Manager on papers submitted after December 6th, 2016. Please ensure that you have an ORCID iD and that it is validated in Editorial Manager. To do this, go to ‘Update my Information’ (in the upper left-hand corner of the main menu), and click on the Fetch/Validate link next to the ORCID field. This will take you to the ORCID site and allow you to create a new iD or authenticate a pre-existing iD in Editorial Manager.**

**Response:** Thank you for your suggestion.The corresponding author's ORCID iD has been validated in Editorial Manager.

6. **We notice that your supplementary figures are uploaded with the file type 'Figure'. Please amend the file type to 'Supporting Information'. Please ensure that each Supporting Information file has a legend listed in the manuscript after the references list.**

**Response:** Thank you for your suggestion.The pictures in our manuscript are all uploaded in the Figure Legend file, so we do not involve supplementary materials

**-Reviewer 1**

**The experiment had been conducted with a small sample sizes, but this limitation is underlyned. Data were analyzed with an appropriate statistical method. There is an error in Line 45: "n recent years..", it misses a letter.**

**Response:** Thank you for your suggestion.We acknowledge that the sample size in this study is small, and this limitation has been explicitly addressed in the discussion section of the manuscript. We have thoroughly discussed the potential impact of the small sample size on the study results and have applied rigorous statistical methods to mitigate this factor as much as possible during the analysis. Despite the limited sample size, the statistical analysis methods used, such as t-tests and binary logistic regression, are reasonable and appropriate, effectively supporting the study's conclusions. Additionally, we have corrected the spelling error in Line 45, changing "n recent years.." to "In recent years.".The corresponding revision is on **Page 3, Line 45**.

**-Reviewer 2**

**This is a very important study and well presented. Kindly find my comments below:**

1. **Introduction:**

**–Line 45 ：I in “In” is missing.**

**Response:** Thank you for your suggestion.The spelling error in Line 45 has been corrected, changing "n recent years.." to "In recent years..".The corresponding revision is on **Page 3, Line 45**.

-**Line 65 ：Change the tense of the objective from future to past.**

**Response:** Thank you for your suggestion.The tense of the objective in Line 65 has been changed from future to past to ensure consistency with the rest of the manuscript.The corresponding revision is on **Page 4, Line 65-67**.

2. **Methods:**

**-Since this is a retrospective analysis, begin the methods with a study design section to introduce readers to the kind of study being conducted. Provide a brief information about the setting where the study was conducted.**

**Response:** Thank you for your suggestion. Given that this study is a retrospective analysis, we have added a "Study Design" section at the beginning of the Methods section to introduce the study type, data sources, and the study time frame, and to provide a brief explanation of the background of the study.The corresponding revision is on **Page 4, Line 70-75**.

**-Change the tense of the section “Study subjects” to past.**

**Response:** Thank you for your suggestion.We have changed the tense of the section “Study subjects” to past.The corresponding revision is on **Page 4-5, Line 76-82**.

**-Line 75 – Delete “absolutely”.**

**Response:** Thank you for your suggestion.We have deleted “absolutely”.

**-Line 76 – Delete third exclusion point. It is just opposite of an inclusion criterion.**

**Response:** Thank you for your suggestion.We have deleted third exclusion point.

**-Line 79-80 – This looks like a repetition of the previous sentence.**

**Response:** Thank you for your suggestion.Indeed, we found that this sentence is repetitive with the previous content. We have already mentioned the relevant information in the "Study Design" section; therefore, we have deleted the redundant content.

**-You don’t need to provide the gender distribution here.**

**Response:** Thank you for your suggestion.We have deleted “gender distribution”

**-Was there a rationale for the 7:3 ratio? Kindly provide that information if there is. If that ratio was adopted from the literature, state and reference.**

**Response:** Thank you for your suggestion.Regarding the use of the 7:3 split ratio, we selected this proportion based on several high-quality studies in the field of radiomics. The 7:3 ratio is widely used for training and validation in radiomics models. The primary purpose is to balance model training and testing, ensuring the model has sufficient data to learn while retaining a portion of data for validation, which helps mitigate the risk of overfitting. Additionally, we reviewed literature focused on the partitioning of training and test sets, and supplemented our research with high-quality references, as indicated in [22-23]. The corresponding references are in **Page 17, Line 283-287**.

**-Lin 99 – Type full form of ROI in title.**

**Response:** Thank you for your suggestion.We haved given the full form of ROI（Regions of interest） in level2 Heading.The corresponding references are in **Page 6, Line 103**.

3. **Results**

**-Line 135 – You have talked about the ratio in the methods. I don’t think you need to repeat here.**

**Response:** Thank you for your suggestion. We haved deleted the radio.

**-T-test and all other univariate statistical analyses used a significance level of 0.05. If that is the case, you need to report the variable where participants in training set were different at baseLine before Table 1.**

**Response:** Thank you for your suggestion. The variables with statistical significance in the training set have been described in the baseline characteristics. The corresponding revised content can be found on **Page 7, Line 140-141**.

**-Line 142-143–“Binary logistic regression….test set”,. Delete the sentence. Already reported in the methods. Focus only on the results and avoid repeating methods in the results.**

**Response:** Thank you for your suggestion. We have deleted the sentence of “Binary logistic regression….test set”.

4. **Discussion**

**-Line 177-190: I think that is too much information for introducing the objective of the study. Trim it down, or better still, delete that section and replace with just a few comprehensive sentences.**

**Response:** Thank you for your suggestion. We have condensed that section, and the corresponding revised content can be found on **Page 12-13, Line 181-187**.

Thank you for your valuable input, which has helped us improve the quality of our figures and the overall reading experience.
